# High-throughput calculations of catalytic properties of bimetallic alloy surfaces

**DOI:** 10.1038/s41597-019-0080-z

**Published:** 2019-05-28

**Authors:** Osman Mamun, Kirsten T. Winther, Jacob R. Boes, Thomas Bligaard

**Affiliations:** 1 SUNCAT Center for Interface Science and Catalysis, SLAC National Accelerator Laboratory, 2575 Sand Hill Road, Menlo Park, California 94025 United States; 2 SUNCAT Center for Interface Science and Catalysis, Department of Chemical Engineering, Stanford University, Stanford, California 94305 United States

**Keywords:** Computational chemistry, Materials for energy and catalysis, Cheminformatics

## Abstract

A comprehensive database of chemical properties on a vast set of transition metal surfaces has the potential to accelerate the discovery of novel catalytic materials for energy and industrial applications. In this data descriptor, we present such an extensive study of chemisorption properties of important adsorbates - e.g., C, O, N, H, S, CH_x_, OH, NH, and *S*H - on 2,035 bimetallic alloy surfaces in 5 different stoichiometric ratios, i.e., 0%, 25%, 50%, 75%, and 100%. To our knowledge, it is the first systematic study to compile the adsorption properties of such a well-defined, large chemical space of catalytic interest. We propose that a collection of catalytic properties of this magnitude can assist with the development of machine learning enabled surrogate models in theoretical catalysis research to design robust catalysts with high activity for challenging chemical transformations. This database is made publicly available through the platform www.Catalysis-hub.org for easy retrieval of the data for further scientific analysis.

## Abstract (summary)

**Table:** 

Design Type(s)	chemical reaction data analysis objective • modeling and simulation objective
Measurement Type(s)	Chemical Properties
Technology Type(s)	digital curation
Factor Type(s)	purity • ratio • Material
Sample Characteristic(s)

Machine-accessible metadata file describing the reported data (ISA-Tab format)

## Background & Summary

Electronic structure calculations from Density functional theory (DFT)^1,2^ is a well established approach for predicting a large range of material properties^3^. In the field of heterogeneous catalysis and electrocatalysis, DFT has provided a deeper understanding of catalytic activity and reaction mechanisms^4^, and has guided the exploration of new catalytic materials. Importantly, the adsorption energy of chemical species to the surface obtained from DFT, has been found to be a strong descriptor for catalytic activity of surfaces^5,6^.

Chemical reactions of interest for sustainable energy applications, including the conversion of CO_2_ and syngas to carbon-based fuels^7,8^, fuel-cell operation^9,10^, and electrochemical water splitting^11^, noble metals such as Pt, Ru, Ag, Ir and Cu are the most active materials. However, a key challenge for large-scale sustainable energy technologies is to identify catalytic materials that are also of high abundance and low cost. In this search, it is instructive to investigate metal alloys, which span a vast set of materials, with the potential to mimic the catalytic properties of the highly active pure metals. Several bimetallic alloys with high catalytic activity have already been identified, including CoMo^12^, BiPt^13^ and Pt-lanthanide alloys such as Pt_3_Y^14^.

**Fig. 1:**
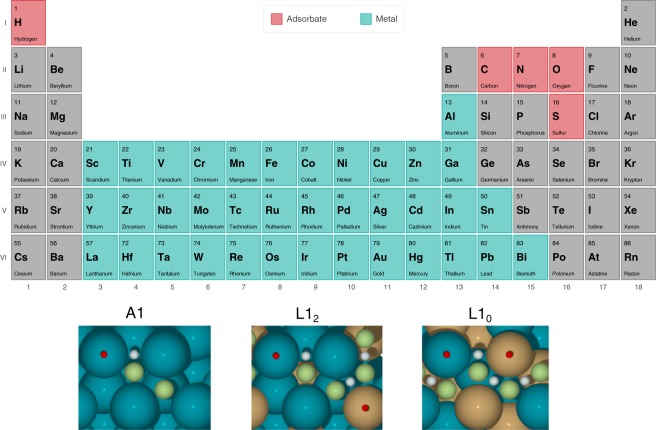
The periodic table outlining five adsorbate elements and the 37 metals included in the dataset. This includes six metals from group 13–15, 17 transition metals, and Lanthanum. Surface geometry and enumerated adsorption sites for the three structures are provided in the lower panel, where top, bridge, and hollow sites are shown in red, white, and green, respectively.

Here, we present a large-scale DFT study of chemical adsorption and hydrogenation on 1,998 bimetallic alloy and 37 pure metal surfaces. Consisting of more than 90,000 systematic DFT calculations, this dataset is intended for machine learning model generation. The alloys were chosen by combining 37 selected metals and transition metals (outlined in the periodic table in Fig. 1) to form alloys in the L1_2_ and L1_0_ Strukturbericht designation, which corresponds to face-centered cubic crystal structures with A_3_B and AB stoichiometries, respectively. The 37 pure metals in the A1 (FCC) structure were included in addition to the 1,998 bimetallic alloys resulting from all possible combinations, such that stoichiometric A:B ratios of 0%, 25%, 50%, 75% and 100% are sampled. The metal surfaces were modeled by cleaving three-layer slabs with a (111) termination for A1 and L1_2_ and a (101) termination for L1_0_, although this termination is also referred to as the (111) miller index^15,16^ when cleaved from the cubic bulk unit cell which is not the standard conventional form.

Atomic adsorption of H, C, N, O, and S was studied for all 2,035 surfaces. In order to systematically sample the adsorption energies, all unique adsorption sites were considered. The unique sites for each of the surface structures are shown in Fig. 1 where the number of sites are 4, 9, and 10 for the A_1_, L1_2_, and L1_0_ surfaces respectively. This gives a total of 96,015 unique surfaces, adsorbate and site combinations (including the empty slabs), where roughly 65,000 calculations are completed. Also, the adsorption of the hydrogenated species CH, NH, CH_2_, CH_3_, SH, OH and H_2_O has been studied for a smaller subset of alloy surfaces, where alloys formed from 16 metals of particular interest for catalysis have been chosen, with approximately 25,000 calculations completed. We note that due to reorientation of adsorbates during structure relaxation, the number of unique surface structures are lower than the number of initially sampled configurations. More than 90,000 adsorption and reaction energies have been parsed from the dataset, where approximately 30,000 adsorption energies stems from the monoatomic adsorbates (H, C, N, O and S), and 10,000 adsorption and reaction energies stems from the hydrogenated adsorbates. The remaining reaction energies are generated by decomposing a set of gas phase molecules of interest for catalysic applications, such as CH_4_(g), NH_3_(g), CO_2_(g), CH_2_CH_2_(g), CH_3_OH(g), H_2_O_2_(g), CH_3_COOH(g), into their atomic constituents on the surfaces.

The dataset is made available from the open repository Catalysis-Hub.org^17^, where reaction energies and barriers from more than 50 publications and datasets can be accessed.

**Fig. 2:**
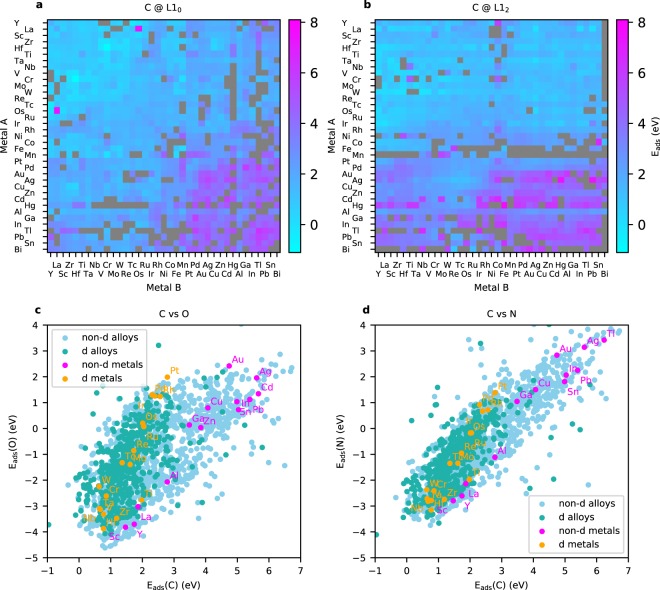
Adsorption energies of selected atomic species. In (**a**,**b**) the C adsorption energy is shown for the 666 L1_0_ (AB) and 1332 L1_2_ (A_3_B) alloy surfaces respectively. Results for the 37 pure metals are shown along the diagonal. Adsorption energies of atomic (**c**) O and (**d**) N are plotted with respect to the C adsorption energy for all materials. References are taken with respect to the reactions: CH_4_(g) − 2 H_2_(g) + * → C*, 0.5 N_2_(g) * → N* and H_2_O(g) − H_2_(g) + * → O* with all species adsorbed to their lowest energy site.

Examples of calculated adsorption energies are given in Fig. 2, showing the results for the most stable adsorption sites for atomic carbon (C), oxygen (O), and nitrogen (N). In Fig. 2(a,b) the adsorption energies are plotted as a function of metal A and B, that are arranged on an improved Pettifor scale^18,19^, with small adjustments for magnetic elements, which ensures a smooth variation of the adsorption energies with composition. Grey areas in the figure can be seen for structures where converged adsorption energies could not be obtained due to surface reconstruction, mismatch in the magnetic structure of the slab and the adsorbate-slab structure or convergence problems for the electronic structure calculation.

Another approach for visualizing adsorption energy trends is to plot the adsorption energy of two adsorbates against each other, which often gives rise to linear scaling relationships for similar surface geometries. Utilizing scaling relationships is a well established approach in theoretical catalysis to model and understand catalytic activity and selectivity^6,20^. In the lower panel of Fig. 2 the correlation between the adsorption of carbon with (c) oxygen and (d) nitrogen is shown. Metals containing a partially filled *d*-band versus a filled or empty *d*-band is labeled as *d*- and *non-d* metals respectively. All alloys containing a *non-d* metal are labeled as *non-d* alloys. While a close to linear relationship between the adsorption of C and O can be seen for the *d* and *non-d* pure metals separately, the correlation between the atomic adsorption energies on the alloys are more complicated, emphasizing the need for more sophisticated methods for modelling these systems, such as data-driven approaches. A link to the script used to plot Fig. 2 by fetching the data directly with the Catalysis-Hub Python API is provided in the *Methods* section.

## Methods

Adsorption energies were calculated with DFT and obtained from the equation:

1 $${E}_{{\rm{a}}{\rm{d}}{\rm{s}}}={E}_{{\rm{s}}{\rm{l}}{\rm{a}}{\rm{b}}+{\rm{a}}{\rm{d}}{\rm{s}}{\rm{o}}{\rm{r}}{\rm{b}}{\rm{a}}{\rm{t}}{\rm{e}}}^{{\rm{D}}{\rm{F}}{\rm{T}}}-{E}_{{\rm{s}}{\rm{l}}{\rm{a}}{\rm{b}}}^{{\rm{D}}{\rm{F}}{\rm{T}}}-\sum _{i}{\alpha }_{i}{E}_{{\rm{i}},{\rm{g}}{\rm{a}}{\rm{s}}}^{{\rm{D}}{\rm{F}}{\rm{T}}},$$

where the gas phase species are chosen among CH_4_(g), H_2_(g), N_2_(g), H_2_O(g) and H_2_S(g). A full list of studied adsorbates and references used are given in Table 1.

**Table 1:** Studied adsorbates listed together with the choice for gas phase reference used to calculate the adsorption energy, Eq. 1.

Adsorbate	Gas phase references
H*	0.5 H_2_(g)
N*	0.5 N_2_(g)
C*	CH_4_(g) − 2 H_2_(g)
O*	H_2_O(g) − H_2_(g)
S*	H_2_S(g) − H_2_(g)
*CH	CH_4_(g) − 1.5 H_2_(g)
*CH_2_	CH_4_(g) − H_2_(g)
*CH_3_	CH_4_(g) − 0.5 H_2_(g)
*NH	0.5 N_2_(g) + 0.5 H_2_(g)
*OH	H_2_O(g) − 0.5 H_2_(g)
H_2_O*	H_2_O(g)
*SH	H_2_S(g) − 0.5 H_2_(g)

The ‘*’ symbol in the adsorbate formula specifies which atom of the molecule binds to the surface.

The Catalysis Kit (CatKit)^21^ software was used to generate the slab structures from optimized bulk systems and to systematically enumerate all high-symmetry adsorption sites. The generated structures were stored and handled with the ASE database^22^.

DFT calculations were performed in the Quantum Espresso (QE) electronic structure code^23^, using the BEEF-vdw exchange correlation functional^24^, a 500 eV plane-wave cutoff, and a 5,000 eV density cutoff. Monkhorst-Pack *k*-point grids of (12, 12, 12) for bulk and (6, 6, 1) for slab calculations were used, with a 0.15 eV Fermi smearing. Spin-polarized calculations were performed only for alloys containing Fe, Ni, Co, and Mn, while allowing the magnetic moments to converge during the electronic structure optimization. Initial magnetic moments of 3, 3, 2, 1 μ_B_ was chosen for Fe, Mn, Co and Ni respectively, and set to zero for all other elements. For the A_1_ and L1_2_ structures, lattice constants were obtained from bulk alloy calculations with an equation of state combined with an energy minimization in QE. For *L*1_0_ structures we used a variable cell optimization in QE with a high plane wave cutoff (800 eV) and then used the resulting lattice constants as initial guess for the final energy minimization with respect to lattice constant parameters - i.e., ‘a’ and ‘c’ - using the Scipy fmin optimizer^25^. Slab geometries were optimized by fixing the two bottom layers and allowing the top layer and adsorbates to relax. Due to the large number of calculations, job submissions were handled with FireWorks^26^ and the CatFlow submodule of CatKit, that provides a FireWorks interface to QE and other electronic structures calculators supported by ASE.

Upon relaxation we found that reconstructions of the metal surfaces, e.g. horizontal sliding or dissociation of the top layer from the slab, are quite common. Also, we found that the adsorbates often reorient into other sites. The relaxed geometries were therefore post-processed with a tailored classification method to label reconstructed surfaces and reclassify the adsorption sites. Only non-reconstructed surfaces have been used to generate adsorption energies, although, as the reconstructed structures could be of interest for model generation, the atomic structures are still made available via the web and python APIs discussed in the *Usage Notes* section.

## Data Records

All the DFT calculations stems from one dataset, generated by O. Mamun, K. Winther, and J. Boes, in the group of Thomas Bliggard at the SUNCAT-Center for Interface Science and Catalysis. The data are made available from two platforms, where the open electronic structure database Catalysis-Hub.org^17^, is the main resource for easy access to parsed adsorption energies. The dataset has been assigned its own permanent link at https://www.catalysis-hub.org/publications/MamunHighT2019. The Catalysis-Hub web interface enables in-browser search for reactions and chemical compositions, with a visualization of atomic geometries, that can be downloaded in several formats including CIF, JSON, VASP POSCAR and Quantum ESPRESSO input. A description on how to download reaction energies and atomic structures with the Catalysis-Hub (CatHub) Python API, available from the Zenodo repository^27^, is provided in the *Usage Notes* section.

In addition, all the raw text output files from the Quantum Espresso calculations have been uploaded to the Materials Cloud archive^28^. The output files can be downloaded and inspected with any text editor, or opened with ASE^22^ to create Atoms objects containing the atomic structures and the results of the calculations.

## Technical Validation

To ensure the quality of the adsorption properties reported herein, the convergence with respect to all calculation parameters have been carefully checked. Adsorption and reaction energies have only been included for surface structures that do not undergo reconstruction upon relaxation. In the case of magnetic surface structures, we have only parsed adsorption and reaction energies if the discrepancy in total magnetization between the empty surface and the surface with the adsorbate is less than 4 in atomic units.

To illustrate the validity of the data, we compare the lattice constant reported in reputed journal articles to the computed lattice constant. We found excellent agreement between our results and previously computed lattice constants which are presented in Tables 2, 3 and 4. In Table 5, we also show a comparison between previously computed adsorption energies to those reported in this article. We also see good agreement between the reported and the computed adsorption energies with slight deviation which may be an artifact of different calculation setup and/or system size, i.e., pseudopotential, smearing scheme, number of layers and lateral size of the slab. For example, the differences in adsorption energies between this work and ref.^8^, which is also available at https://www.catalysis-hub.org/publications/SchumannSelectivity2018, can be attributed to the use of a 4 layer (3 × 3) repeated surface slab model, compared to the 3 layer (2 × 2) slab used in this study.

**Table 2:** Equilibrium lattice constant of pure FCC metals, in Å.

	Computed lattice constant [a]	Experimental lattice constant [a]
*Pb*	4.89	4.91^29^
*Ni*	3.53	3.51^29^
*Pt*	3.95	3.91^29^
*Ir*	3.88	3.84^30^
*Al*	4.02	4.02^29^
*Cu*	3.67	3.60^29^
*Pd*	3.99	3.88^29^
*Au*	4.21	4.06^29^
*Ag*	4.22	4.06^29^

**Table 3:** Equilibrium lattice constant of *L*1_2_ metals, in Å.

	Computed lattice constant [a]	Reported lattice constant [a]
*Pd*_3_ *Co*	3.88	3.93^31^
*Pd*_3_ *Ni*	3.89	3.93^31^
*Fe*_3_ *Pt*	3.72	3.74^16^
*Ni*_3_ *Pt*	3.67	3.66^16^
*Zr*_3_ *Al*	4.38	4.37^32^
*Sc*_3_ *Al*	4.41	4.42^32^

**Table 4:** Equilibrium lattice constant of *L*1_0_ metals, in Å.

	Computed lattice constant [a]	Computed $[\frac{{\boldsymbol{c}}}{{\boldsymbol{a}}}]$	Reported lattice constant [a]	Reported $[\frac{{\boldsymbol{c}}}{{\boldsymbol{a}}}]$
*PdCo*	3.80	0.98	3.83^31^	0.98^31^
*PdNi*	3.87	0.94	3.83^31^	0.95^31^
*PtFe*	3.88	0.97	3.86^16^	0.97^16^
*PtNi*	3.86	0.94	3.85^16^	0.95^16^
*CoPt*	3.83	0.97	3.83^33^	0.97^33^

**Table 5:** Adsorption energies of various adsorbates on pure metals in eV.

Reaction	Metal	Computed adsorption energy [eV]	Reported adsorption energy [eV]
0.5 *H*_2_(*g*) + * → H*	*Pt*	−0.31	−0.24^8^
0.5 *H*_2_(*g*) + * → H*	*Pd*	−0.29	−0.28^34^
0.5 *H*_2_(*g*) + * → H*	*Re*	−0.51	−0.60^34^
0.5 *H*_2_(*g*) + * → H*	*Rh*	−0.28	−0.34^35^
0.5 *H*_2_(*g*) + * → H*	*Ir*	−0.16	−0.19^8^
0.5 *H*_2_(*g*) + * → H*	*Ag*	0.34	0.44^35^
0.5 *H*_2_(*g*) + * → H*	*Cu*	−0.05	0.03^35^
*O*_2_(*g*) + * → 2O*	*Pt*	−1.90	−1.96^35^
*H*_2_*O*(*g*) + * → OH* + 0.5 *H*_2_(*g*)	*Pd*	0.66	0.60^8^
*H*_2_*O*(*g*) + * → OH* + 0.5 *H*_2_(*g*)	*Co*	−0.24	−0.31^8^
*H*_2_*O*(*g*) + * → OH* + 0.5 *H*_2_(*g*)	*Ag*	0.72	0.63^8^
*H*_2_*O*(*g*) + * → OH* + 0.5 *H*_2_(*g*)	*Cu*	0.19	0.28^8^
*H*_2_*O*(*g*) + 2* → OH* + H*	*Ag*	1.02	1.13^8^
*H*_2_*O*(*g*) + 2* → OH* + H*	*Cu*	0.28	0.36^8^
*CH*_3_ * + * → *CH*_2_ * + H*	*Ag*	1.87	2.11/1.89^8,36^
*CH*_3_ * + * → *CH*_2_ * + H*	*Cu*	0.92	1.15/0.94^8,36^
*CH*_3_ * + * → *CH*_2_ * + H*	*Au*	0.71	0.77^36^
0.5 *N*_2_(*g*) + * → N*	*Pd*	1.06	0.94^34^
0.5 *N*_2_(*g*) + * → N*	*Re*	−1.33	−1.46^34^
0.5 *N*_2_(*g*) + * → N*	*Pt*	0.78	0.91^34^
0.5 *N*_2_(*g*) + * → N*	*Ir*	0.34	0.30^34^
0.5 *N*_2_(*g*) + * → N*	*Rh*	0.05	0.06^34^
0.5 *N*_2_(*g*) + * → N*	*Au*	2.80	2.92^34^
0.5 *N*_2_(*g*) + * → N*	*Ru*	−0.65	−0.57^34^
0.5 *N*_2_(*g*) + * → N*	*Ag*	3.11	3.19^34^
0.5 *N*_2_(*g*) + * → N*	*Cu*	1.50	1.53^34^

The raw DFT reaction energy without energy corrections is reported for all values in the table. (Note in ref.^8^ a +0.1 eV correction was applied to H_2_(g)).

## Usage Notes

The CatHub software module, which is available from the Zenodo repository^27^, provides a Python API which is better suited for fetching a large amount of data from the Catalysis-Hub repository. A small script for obtaining pre-parsed adsorption energies in Python is provided below:

from cathub.query import get_reactions

get_reactions(pubId = ‘MamunHighT2019’,

n_results = 2,

surfaceComposition = ‘Mo + Ru’,

reactants = “CH4gas + H2”,

sites = “~hollow”,

products = ‘C’)

which returns a JSON dictionary on the form:

{‘reactions’:

{‘edges’: [x‘

{‘node’:

{‘Equation’: ‘CH4(g) - 2.0H2(g) + * - > C*‘,

‘activationEnergy’: None,

‘chemicalComposition’: ‘Mo3Ru9’,

‘coverages’: {‘C’: 0.25},

‘dftCode’: ‘Quantum ESPRESSO 5.1’,

‘dftFunctional’: ‘BEEF-vdW’,

‘facet’: ‘111’,

‘products’: {‘Cstar’: 1},

‘pubId’: ‘MamunHighT2019’,

‘reactants’: {‘star’: 1, ‘H2gas’: -2.0,

‘CH4gas’: 1.0}’,

‘reactionEnergy’: 1.2068607934897955,

‘sites’: {‘C’: ‘hollow|A_A_A|HCP’},

‘surfaceComposition’: ‘Ru3Mo’}

},

{‘node’:…}

],

‘totalCount’: 10}

}

}.

Note that each data entry is given as a ‘node’ in a list of ‘edges’, utilizing the graph-theory based query language GraphQL (https://graphql.org/). Since the Catalysis-Hub repository contains several datasets from different publications, the “pubId = ‘MamunHighT2019’” argument must be assigned in the script above in order to query only this dataset. The script above queries for entries with hollow-site adsorption of C (product) with respect to the relevant gas phase species (reactants), on surfaces containing Mo as well as Ru. The reaction and product entries must be chosen (and matched) among the adsorbates and gas phase references in Table 1. A more specific query for adsorption site can be made by using the site names specified in Table 6.

**Table 6:** Names of sampled adsorption sites, where A and B refers to the choice of metals. The sites marked with ‘*’ have not been sampled with the initial configuration shown in Fig. 1, but stems from deviation from the hexagonal surface structure for some of the L1_0_ alloys or reorientation of the adsorbate into a subsurface site.

A1	L1_2_	L1_0_
top|A	top|A	top|A
	top|B	top|B
bridge|A_A|A	bridge|A_A|A	bridge|A_A|A*
		bridge|B_B|B*
	bridge|A_A|B	bridge|A_A|B
		bridge|B_B|A
	bridge|A_B|A	bridge|A_B|A
		bridge|A_B|B
hollow|A_A_A|HCP	hollow|A_A_A|HCP	
hollow|A_A_A|FCC	hollow|A_A_A|FCC	
	hollow|A_A_B|HCP	hollow|A_A_B|HCP
	hollow|A_A_B|FCC	hollow|A_A_B|FCC
		hollow|A_B_B|HCP
		hollow|A_B_B|FCC
subsurface*	subsurface*	subsurface*
		4fold|A_A_B_B*

Furthermore, easy access to all the atomic structures, calculation results and parameters in the study, can be obtained with the ASE database interface^22^, where the CatHub module features a convenient wrapper around the ASE db command line interface (CLI), used directly from a terminal. For example, the query:

cathub ase ‘pub_id = MamunHighT2019, relaxed = 1’

will return a list with the first 20 results (out of approximately 90,000) for the relaxed configurations in the study. The initial geometries can be queried by setting ‘relaxed = 0’. The atomic structures are labeled with an several key-value-pair metadata, that can be used to query the dataset. For example:

cathub ase ‘Pt,pub_id = MamunHighT2019,relaxed = 1,

reconstructed = 0,SB_symbol = L10

-c formula,energy,adsorbate,site,site_type -L 100’

--gui

will return the 100 first relaxed and non-reconstructed structures containing Pt in the L1_0_ structure, as a table containing the chemical formula, the DFT total energy, the name of the adsorbate, and site information as well as opening all the matching structures in the ASE gui visualizer. A description of ASE native columns as well as special key value pairs assigned in this study is given in Table 7. We refer to ref.^17^ for a detailed description of the Catalysis-Hub database structure.

**Table 7:** Data structure for storing atomic structures with the ASE database. The upper panel show the native ASE database columns and the lower panel dataset specific key value pairs.

Key	Description	Datatype
id	Local database id	int
unique_id	Globally unique hexadecimal id	str
ctime	Creation time	float
mtime	Modification time	float
user	User name	str
numbers	Atomic numbers	int
pbc	Periodic boundary condition flags	bool
cell	Unit cell	float
positions	Atomic positions	float
initial_magmoms	Initial atomic magnetic moments	float
initial_charges	Initial atomic charges	float
masses	Atomic masses	float
tags	Tags	int
momenta	Atomic momenta	float
constraints	Constraints	list of dict
energy	Total energy	float
forces	Atomic forces	float
stress	Stress tensor	float
dipole	Electrical dipole	float
charges	Atomic charges	float
magmom	Magnetic moment	float
magmoms	Atomic magnetic moments	float
calculator	Calculator name	str
calculator_parameters	Calculator parameters	dict
metalA	Name of A metal (see Fig. 1)	str
metalB	Name of B metal (see Fig. 1)	str
slab_name	Chemical stiochemitry of slab	str
SB_symbol	Structurbericht designation	str
adsorbate	Adsorbate (see Table 1)	str
fw_id	User specific Fireworks job id	int
geometry	Unique structure descriptor	str
pub_id	Dataset id (MamunHighT2019)	str
reconstructed	Surface reconstruction	bool (0 or 1)
relaxed	Relaxed structure	bool (0 or 1)
site	Primary site descriptor	str
site_type	Secondary site descriptor	str
state	State (bulk, molecule or slab)	str

## ISA-Tab metadata file

Download metadata file

## Data Availability

The CatHub python API and the CatKit software packages are available open-source from the GitHub repository at https://github.com/SUNCAT-Center/. In addition, the latest stable version of the CatHub module is available from the Zenodo repository^27^. The Python scripts used for plotting the data shown in Fig. 2 is made available as a tutorials at https://github.com/SUNCAT-Center/CatHub/tree/master/tutorials/1_bimetallic_alloys/. The code used to classify the adsorption sites is made available at https://github.com/SUNCAT-Center/CatHub/tree/master/cathub/classification.py.
